# Soil Depth Significantly Shifted Microbial Community Structures and Functions in a Semiarid Prairie Agroecosystem

**DOI:** 10.3389/fmicb.2022.815890

**Published:** 2022-06-08

**Authors:** Zineb Rchiad, Mulan Dai, Chantal Hamel, Luke D. Bainard, Barbara J. Cade-Menun, Yves Terrat, Marc St-Arnaud, Mohamed Hijri

**Affiliations:** ^1^African Genome Center, Mohammed VI Polytechnic University, Ben Guerir, Morocco; ^2^Institut de Recherche en Biologie Végétale, Département de Sciences Biologiques, Université de Montréal and Jardin Botanique de Montréal, Montréal, QC, Canada; ^3^Research and Development of Enterra Corporation, Vancouver, BC, Canada; ^4^Quebec Research and Development Centre, Agriculture and Agri-Food Canada, Québec, QC, Canada; ^5^Swift Current Research and Development Centre, Agriculture and Agri-Food Canada, Swift Current, SK, Canada; ^6^Agassiz Research and Development Centre, Agriculture and Agri-Food Canada, Agassiz, BC, Canada

**Keywords:** metagenomic analysis, soil depth, soil microbial community, soil functions, semiarid grassland, North American Great Plains

## Abstract

The North American Great Plains cover a large area of the Nearctic ecozone, and an important part of this biome is semiarid. The sustainable intensification of agriculture that is necessary to produce food for an ever-increasing world population requires knowledge of the taxonomic and functional structure of the soil microbial community. In this study, we investigated the influence of soil depth on the composition and functions of the microbial communities hosted in agricultural soils of a semiarid agroecosystem, using metagenomic profiling, and compared them to changes in soil chemical and physical properties. Shotgun sequencing was used to determine the composition and functions of the soil microbial community of 45 soil samples from three soil depths (0–15 cm, 15–30 cm, and 30–60 cm) under different agricultural land use types (native prairie, seeded prairie, and cropland) in southwest Saskatchewan. Analysis of community composition revealed the declining abundance of phyla Verrucomicrobia, Bacteroidetes, Chlorophyta, Bacillariophyta, and Acidobacteria with soil depth, whereas the abundance of phyla Ascomycota, Nitrospirae, Planctomycetes, and Cyanobacteria increased with soil depth. Soil functional genes related to nucleosides and nucleotides, phosphorus (P) metabolism, cell division and cell cycle, amino acids and derivatives, membrane transport, and fatty acids were particularly abundant at 30–60 cm. In contrast, functional genes related to DNA and RNA metabolism, metabolism of nitrogen, sulfur and carbohydrates, and stress response were more abundant in the top soil depth. The RDA analysis of functional genes and soil physico-chemical properties revealed a positive correlation between phages and soil organic P concentrations. In the rooting zone of this semiarid agroecosystem, soil microbes express variable structural patterns of taxonomic and functional diversity at different soil depths. This study shows that the soil microbial community is structured by soil depth and physicochemical properties, with the middle soil depth being an intermediate transition zone with a higher taxonomic diversity. Our results suggest the co-existence of various microbial phyla adapted to upper and lower soil depths in an intermediate-depth transition zone.

## Introduction

Southwest Saskatchewan is a semiarid region of the North American Great Plains, an important component of the Nearctic ecozone. According to the Köppen climate classification, this ecoregion generally experiences a semiarid climate, with about 300–380 mm of annual precipitation (Kottek et al., 2006) two-thirds of which occurs in the growing season (Anderson and Cerkowniak, 2010). Brown Chernozem, Dark Brown, and Black Chernozem soils (Soil Classification Working Group, 1998) have evolved in this ecoregion spanning 638,400 km^2^ (Primm et al., 2016). In the semiarid prairie ecoregion, both soil properties and human influences on soil are determined by the traits of the climate (Anderson and Cerkowniak, 2010). More than 85% of the prairie ecoregion is pasture and croplands (Primm et al., 2016; Nelkner et al., 2019), and management practices (e.g., summer fallow, soil tillage, weed control, overgrazing, fertilization) will influence soil chemical and physical properties and may cause erosion (Anderson and Cerkowniak, 2010; Zhang et al., 2015; Cade-Menun et al., 2017).

Soils are stratified and soil depth is a key factor influencing biotic and abiotic soil parameters, such as carbon (C) and nitrogen (N) concentrations (Baisden et al., 2002) and microbial diversity (Frank-Fahle et al., 2014) which declines beyond the soil surface zone corresponding to the location of the root biomass.

Soil depth affects the abundance of soil microorganisms and the structures of their microbial communities (Oehl et al., 2005; Taniguchi et al., 2012; Becerra et al., 2014) and shapes their abilities to contribute to the ecological services of plant production (Koven et al., 2013; Fan et al., 2016; Jiao et al., 2018). Understanding the microbial patterns specific to different soil depths, and the factors shaping them, is essential to better exploit the potential of soil functions and propose new strategies to enhance soil sustainability. For instance, the use of deeply-rooted crops to replace shallow-rooted ones is proposed as a promising natural solution for mitigating the effect of drought and improving soil health (reviewed in Pierret et al., 2016). Therefore, the identification of microbial communities ensuring critical processes for plant roots at varying depths in different agro-ecosystems could assist the selection of adapted crops. Moreover, more information on microbial diversity and abundance at varying soil depths will guide the choice of the relevant depths in soil and root sampling experiments, in addition to numerous fundamental questions related to soil formation and C sequestration, among others.

Multiple microbial taxa share similar and/or complementary molecular pathways, establishing a redundancy in soil functions. Hence, loss of taxonomic diversity does not necessarily impact soil functional diversity (Souza et al., 2015). Therefore, the diversity of soil functional genes can be considered more important than the taxonomic diversity of soil microorganisms when studying soil functions (Burke et al., 2011; Barberán et al., 2014; Zhang et al., 2017). The amount of data available in the literature on the spatial distribution of functional genes with increasing soil depth is by far less than satisfactory. Despite the importance of deep soil layers, most available literature on soil microorganisms focuses on the accessible top soil layer, marginalizing the ecological functions of deeper soil layers and leaving a big gap in our understanding of vertical distribution of soil functions. We have used metagenomics to assess the impact of soil depth and varying soil physical and chemical properties on soil taxonomic and functional microbial profiles.

The objective of this study is to identify the characteristics of soil functions and microbial diversity at three soil depths (0–15, 15–30, and 30–60 cm) in a semiarid ecosystem. Since there are less air, nutrients, roots, and organic matter deeper in soils compared to the surface (Koven et al., 2013; Fan et al., 2016), it is plausible that only the tough and enduring taxa can thrive in deeper soil, reducing diversity as depth increases. Soil samples used in this study are part of a larger project investigating soil properties under different agricultural land uses in southwest Saskatchewan, which showed significant differences in soil chemical and physical properties with depth (Cade-Menun et al., 2017). In light of these changes in physical and chemical properties, we hypothesize that the change in the abundance and diversity of soil functional genes and in the composition of microbial communities with increasing soil depth forms a gradient with a higher diversity in the intermediate soil layer where microbial phyla that are adapted to higher and lower soil depths co-exist.

## Materials and Methods

### Field Description and Soil Sampling

This study was part of a larger project, and full details about the study sites can be found in previous publications (Bainard et al., 2017; Cade-Menun et al., 2017; Liu et al., 2018). Briefly, samples were collected from 15 sites in the Brown soil zone of the North American Great Plains, north of the Canada–United States border (Supplementary Figure 1), between latitude 49.048717° and 49.7304222°, longitude −107.586917° and −108.026867°, and at an altitude between 795 and 955 m above sea level. Soils are Orthic Brown Chernozems in the Canadian classification system (Aridic Kastanozems in the FAO system and Aridic Boroll, in the USDA classification system). Samples were collected from five locations, each with three land use types: grazed native prairie vegetation (native prairie), grazed crested wheatgrass [*Agropyron cristatum* (*L*.) *Gaetern*] pasture (seeded prairie), and cereal-based crop rotations (cropland). At each location, all land use types were within 5 km, with similar texture, topography, and environmental factors. Soil samples were collected between 22 and 30 May 2013 from four 1-m^2^ quadrats situated along a 10-m transect at each study site. One 5-cm diameter soil core was taken from six depths (0–7.5, 7.5–15, 15–30, 30–60, 60–90, and 90–120 cm) in each quadrat using a truck-mounted hydraulic soil coring device (Giddings Machine Company, Windsor, CO, United States). These are deep soils rich in organic matter and calcium carbonate, which provides solid cores by this sampling method, facilitating the sorting of soil depth fractions and minimizing cross-contaminations between soil layers. Soil samples were transported to the laboratory on ice and were homogenized after passing materials through a 2-mm sieve. Metagenomic analysis was conducted on three depths: 0–15 cm, 15–30 cm, and 30–60 cm, with the 0–15-cm samples prepared by combining equal amounts of soil from the 0–7.5- and 7.5–15-cm soil depths (Bainard et al., 2017). Samples were kept at 4°C during manipulation.

### DNA Extraction and Shotgun Sequencing

Total genomic DNA was extracted using the PowerSoil™ DNA Isolation Kit (Qiagen, ON, Canada) from 0.5 g of homogenized soil from each sampling point (i.e., quadrat) and soil depth. The DNA extracts of the quadruplicates from each sampling site were pooled together by depth for a total of 45 composite samples (3 depths × 3 land use × 5 locations). The DNA samples were quantified and metagenomic libraries for shotgun sequencing were prepared using the TruSeq DNA HT Sample Prep Kit. Libraries were sequenced at the Centre d’expertise et de services, Génome Québec (Montreal, QC, Canada) using the Illumina HiSeq 2500 2 × 150 bp (Illumina Inc., San Diego, CA, United States) platform. Samples were multiplexed across 4.5 HiSeq lanes.

### Taxonomic Analysis

After uploading the raw fastq files, data were preprocessed by using SolexaQA (Cox et al., 2010) to trim low-quality regions. The DynamicTrim filter of the SolexaQA software was used to crop each read to its longest contiguous segment for which quality scores are greater than 30. Sequences lower than 75 nucleotides in length were discarded for further analysis.

Raw sequencing data from each sampling site were uploaded and analyzed with the online Meta Genome Rapid Annotation using Subsystem Technology [MG-RAST version 3.3.6 pipeline, Meyer et al. (2008)^1^ ]. The MG-RAST metagenomic server also provided a screening stage to remove the reads that were near-exact matches to publicly available genomes. Taxonomic classification of the sequences was conducted by comparison against the RDP (Ribosomal Databases Project^2^; Wang et al., 2007) and M5NR (M5 Non-Redundant Protein) databases based on the “best hit classification” method. The parameters used were cut-off 60% for identifying protein with a maximum e-value of 1E^–5^ and minimum alignment length of 15. Comparison of the taxonomic annotation results between RDP and M5NR showed lower average percent identity of the hits (≈71%) and shorter average alignment length of the hits (≈42 bp) in M5NR. The average percent identity obtained with RDP was above 99% and the average alignment length was approximately 75 bp. Therefore, we use RDP database for taxonomic analysis in this study.

### Functional Analysis

The functional analysis of these soils at different depths was conducted using the SEED subsystems (Overbeek et al., 2005) on the MG-RAST v.3.3.6 pipeline with the same set of parameters as described for the taxonomic analysis above. The levels of subsystems considered in SEED are: (1) level 1, the highest categories; (2) level 2, the second highest categories; (3) level 3, concrete functional gene clusters; (4) actual individual function of a protein-coding gene. In order to adequately describe relevant functions, reduce calculations, and avoid functional redundancy on individual gene functions, we focused on: level 1, for an overview of the distribution of functional categories, and level 3, for information on the enrichment of functional genes present in the soil under study.

### Diversity Analysis

The metagenomic dataset from MG-RAST was downloaded and submitted to STAMP (Statistical Analysis of Metabolic Profile; Parks et al., 2014) for further analysis. The analysis of the functional and taxonomic community of prairie soil microorganisms was conducted using the relative abundance of subsystem levels and taxa that were generated by STAMP. Datasets of metagenomic relative abundance were read into R (version 3.2.2), and the Shannon diversity index was calculated with the “diversity” function of the “vegan” package.

### Analysis of Soil Chemical and Physical Properties

Analysis of soil physical (bulk density, texture) and chemical properties was conducted for Cade-Menun et al. (2017), and is summarized in the Supplementary Materials. The results from Cade-Menun et al. (2017) for the 0–7.5 and 7.5–15-cm depths were averaged to give values for the 0–15-cm depth.

### Statistical Analysis

Metagenomic datasets were downloaded and submitted to STAMP from MG-RAST to estimate the relative abundance of genes and taxa, as described above. *Post hoc* comparisons were done using the Tukey–Kramer test with 95% confidence intervals. Effect size was measured using Eta-squared values, and Benjamini-Hochberg FDR correction was used in multiple test comparisons. Analysis of variance (ANOVA) was used to test the effect of soil depth on soil functional and taxonomic profiles. Comparisons for all pairs of soil depth used Tukey–Kramer HSD (α = 0.05) to test the difference between means. Non-metric multidimensional scaling (NMDS) plots were used to visualize the distribution of sample clusters in various soil depths based on the functional and taxonomic relative abundance matrix, respectively, generated by STAMP. These plots were generated by R with the function “metaMDS” in the “vegan” package. Permutation-based multivariate analysis of variance (PERMANOVA) (Anderson, 2001), was performed on 45 samples considered for this investigation with the function “Adonis” of the R package vegan v 2.5-6. The “Goodness of fit” draws ordination diagrams with sampling sites to measure the stress. Redundancy analysis (RDA) was conducted in R and used to test the significance of the relationship between soil depth and subsystem leve1. Heatmaps of the abundance of taxonomic phyla and families were prepared using the “heatmap.2” function of the ‘gplots’ package in R (Warnes et al., 2015). Statistical analysis of soil chemical and physical data is described in the Supplementary Materials.

## Results

### Summary of MG-RAST Pipeline

Sequencing of the 45 samples yielded over 5.6E + 8 reads for each soil depth, and >94% of the sequences went through quality control with an average length of 150 base pairs (bp; Table 1). After “best hit classification” against the RDP and SEED subsystems databases, an average of 1.2E + 5 sequences in each soil depth were predicted taxonomic proteins, and 2.5E + 8 sequences were assigned with known functions in SEED (Table 1).

**TABLE 1:** Summary of original sequences and sequences processed through MG-RAST pipeline, and relative abundance of functions on subsystem level 1 at different soil depths. Values are means ± std. err. (*n* = 15).

		Depth		*p*-Value
	0–15 cm	15–30 cm	30–60 cm	
Total reads	567,292,988	582,099,874	576,251,342	
Quality control sequences	536,759,803	553,397,918	545,981,917	
MG-RAST assigned reads (RDP)	135,819	118,480	111,110	
% of MG-RAST assigned reads (RDP)				
Archaea	1.02%	1.17%	0.86%	
Bacteria	91.96%	91.56%	91.47%	
Eukaryota	2.05%	2.53%	2.62%	
Unassigned	4.36%	4.2%	4.47%	
Unclassified sequences	0.62%	0.54%	0.58%	
MG-RAST assigned reads (known functions with SEED)	258,594,964	251,629,816	272,173,086	
**% of MG-RAST assigned reads per sample (subsystems level1**)				
Amino acids and derivatives	11.32(±0.08)*b*	11.4(±0.08)*b*	11.51(±0.12)*a*	**<0.0001**
Carbohydrates	15.9(±0.24)*a*	15.85(±0.24)*ab*	15.67(±0.25)*b*	**0.04**
Cell division and cell cycle	1.06(±0.02)*b*	1.07(±0.02)*b*	1.09(±0.01)*a*	**<0.0001**
Cell wall and capsule	3.6(±0.07)*a*	3.49(±0.09)*b*	3.46(±0.08)*b*	**<0.001**
Clustering-based subsystems	13.82(±0.09)	13.81(±0.12)	13.89(±0.14)	0.18
Cofactors, vitamins, prosthetic groups, pigments	6.5(±0.06)	6.49(±0.06)	6.53(±0.1)	0.26
DNA metabolism	3.78(±0.04)*a*	3.7(±0.03)*b*	3.7(±0.03)*b*	**<0.0001**
Dormancy and sporulation	0.154(±0.003)*a*	0.15(±0.003)*b*	0.151(±0.005)*ab*	**0.047**
Fatty acids, lipids, and isoprenoids	3.74(±0.05)*b*	3.75(±0.04)*b*	3.79(±0.04)*a*	**0.007**
Iron acquisition and metabolism	0.47(±0.06)*ab*	0.45(±0.04)*b*	0.49(±0.03)*a*	**0.046**
Membrane transport	2.15(±0.07)*b*	2.21(±0.06)*ab*	2.25(±0.06)*a*	**<0.001**
Metabolism of aromatic compounds	1.86(±0.04)	1.86(±0.04)	1.85(±0.04)	0.807
Miscellaneous	7.01(±0.05)*a*	6.93(±0.05)*b*	6.91(±0.1)*b*	**0.001**
Motility and chemotaxis	0.71(±0.04)*a*	0.6(±0.03)*b*	0.57(±0.02)*c*	**<0.0001**
Nitrogen metabolism	0.83(±0.01)*a*	0.8(±0.02)*b*	0.8(±0.01)*b*	**<0.0001**
Nucleosides and nucleotides	2.48(±0.03)*c*	2.52(±0.03)*b*	2.56(±0.03)*a*	**<0.0001**
Phages, prophages, transposable elements, plasmids	2.24(±0.51)	2.78(±0.66)	2.75(±0.88)	0.079
Phosphorus metabolism	1.05(±0.04)*c*	1.09(±0.03)*b*	1.11(±0.02)*a*	**<0.0001**
Photosynthesis	0.105(±0.002)*a*	0.1(±0.004)*b*	0.097(±0.004)*c*	**<0.0001**
Potassium metabolism	0.22(±0.01)	0.22(±0.01)	0.22(±0.008)	0.09
Protein metabolism	7.04(±0.08)	7.01(±0.1)	7.05(±0.05)	0.35
Regulation and cell signaling	1.12(±0.02)*a*	1.11(±0.02)*a*	1.1(±0.02)*b*	**<0.0001**
Respiration	2.76(±0.1)*a*	2.74(±0.06)*a*	2.66(±0.04)*b*	**<0.0001**
RNA metabolism	4.52(±0.07)*a*	4.42(±0.06)*b*	4.43(±0.04)*b*	**<0.0001**
Secondary metabolism	0.38(±0.01)	0.38(±0.02)	0.38(±0.01)	0.44
Stress response	2.1(±0.02)*a*	2.02(±0.03)*b*	1.98(±0.02)*c*	**<0.0001**
Sulfur metabolism	1.02(±0.03)*a*	0.99(±0.03)*a*	0.95(±0.03)*b*	**<0.0001**
Virulence, disease and defense	2.06(±0.03)	2.04(±0.05)	2.02(±0.05)	0.1

*RDP, ribosomal database project.*

*Different letters within each row and p-values in bold indicate statistically significant differences (ANOVA followed by Tukey HSD; α = 0.05).*

Exploration of the data with NMDS analysis revealed an influence of soil depth. Both subsystems level 1 functional data and taxonomic data at the family level (Supplementary Figure 2) formed clusters according to soil depth. Using PERMANOVA test, we observed that soil depth revealed significant changes in both taxonomic and functional profiles (*p* = 0.001). The NMDS plot shows minimal overlap of functional gene or taxonomic communities clusters based on soil depth, and stress values indicate that both the functional subsystems level 1 (stress value = 0.16) and taxonomic family level (stress value = 0.15) were very well represented in the reduced dimensions of the plots. This analysis revealed clear differences both in the structure of the microbial community and functional genes assemblage among soil depths. Therefore, further analyses of the taxonomic and functional gene communities among soil depths were made.

### Taxonomic Community in Various Soil Depths

The taxonomic community analyses performed with the RDP database available in the MG-RAST server showed that in all soil depths examined in this study, the majority of the sequences belong to a single bacterial phylum, Actinobacteria, accounting for ∼30% of all quality sequences. Unclassified sequences of Eukaryota, Bacteria, Archaea, and unassigned sequences (unknown sequences showing no similarity with any known sequences in the RDP database) accounted for approximately 31%. Proteobacteria was the second largest group (≈12%) of identified phyla, and Firmicutes was the third (≈7%). All of these dominant phyla belong to the domain of Bacteria. The relative abundance of these most abundants bacterial phyla did not vary with soil depth (Supplementary Table 1).

**FIGURE 1:**
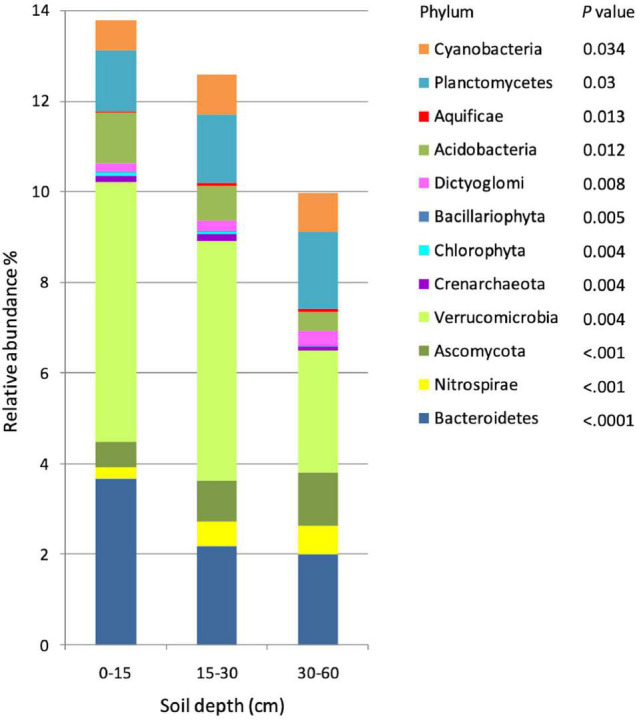
Relative abundance of microbial taxonomic profiles at the phylum level in three soil depths (*n* = 15). Phyla were determined using 16s rRNA fragments against the RDP database, and only significantly different phyla among soil depths are showed in the graph. Differences in phyla among depths are indicated by *p*-values < 0.05.

On the other hand, data analysis revealed significant differences in the taxonomic composition of soil microbial communities among the three soil depths. Communities varied with soil depth at both the phylum and family levels. Overall, 43 phyla were detected, out of which 12 had a distribution significantly affected by soil depth. The most abundant phylum affected by soil depth was Verrucomicrobia, with a relative abundance of about 5% in the 0–15-cm and 15–30-cm soil depths, decreasing to about 2.6% at 30–60 cm (Figure 1). Distribution of Bacteroidetes was 3.6% at 0–15 cm and approximately 2% in the two deeper depths. In domain Eukaryota, Ascomycota was significantly less abundant at 0–15 cm than deeper in the soil (0.9% at 15–30 cm, and 1.18% at 30–60 cm) a result that could be attributed to a decreased dominance. The other significant differences were found in Eukaryota phyla Chlorophyta and Bacillariophyta, which displayed decreasing abundances with soil depth (Figure 1 and Supplementary Table 1). Only one phylum of domain Archaea, Crenarchaeota, displayed a significant variability in relative distribution with soil depth; Crenarchaeota were relatively more abundant at 0–15-cm (0.14%) and 15–30-cm (0.17%) than at the 30–60-cm (0.08%) soil depth.

**FIGURE 2:**
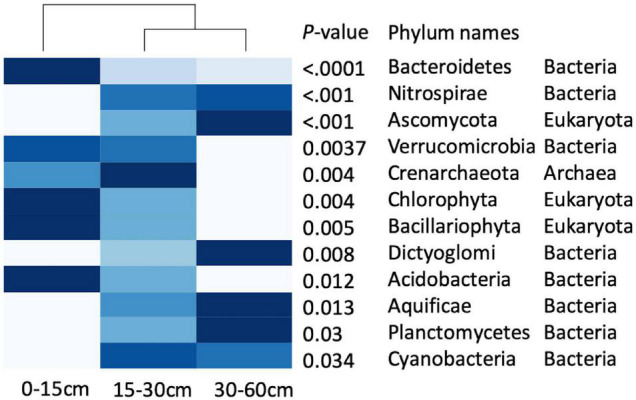
Heatmap of the taxonomic profiles at the phyla level in three soil depths, showing the relative abundances of phyla in each soil depth (*n* = 15), the deeper the blue cell, the higher the relative abundance of functions. Differences in phyla among soil depths are indicated by *p*-values < 0.05.

The family level profiles of the soil microbial communities in the 0–15-cm and in the 30–60-cm soil depths segregated markedly in a principal component analysis (PCA), whereas the profiles of the communities at 15–30 cm were intermediate, with a partial intersection with the microbial profiles at 30–60 cm (Supplementary Figure 3). The heatmaps of the microbial profiles at the phylum/family level show clear differences in the communities established in the top (0–15 cm) and the deepest (30–60 cm) soil depths, while the relative abundance of the microbial phyla at 15–30 cm exhibits an intermediate profile (Figure 2 and Supplementary Figure 4), concomitant with the intermediate state observed in Supplementary Figure 3. Similar patterns of the taxonomic profile variation were found at the family level (Supplementary Figure S4). The Shannon *H*′ diversity index was 2.85 at 0–15-cm, 2.91 at 15–30-cm and 2.83 for the 30–60-cm soil depths. Almost all the prokaryotic phyla differentially distributed in the soil profile were relatively more abundant either in the 0–15-cm or the 30–60-cm depth, with the exception of Crenarchaeota and Cyanobacteria which were relatively most abundant at 15–30 cm. The fungal phylum Ascomycota was more abundant at 30–60 cm, while the other two Eukaryotic phyla, Chlorophyta and Bacillariophyta, were more abundant at 0–15-cm depth. The three dominant bacterial phyla, Bacteroidetes, Verrucomicrobia, and Acidobacteria were more abundant in the top soil depth (Figure 2).

### Functional Metagenome Profiles

**FIGURE 3:**
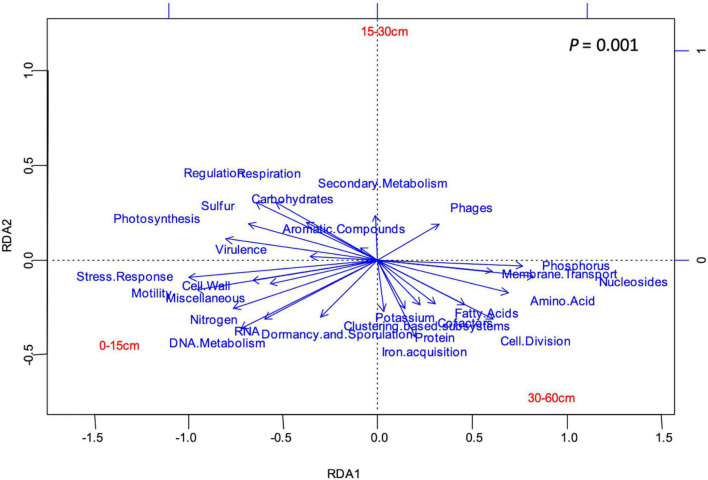
RDA showing the relationship (*p* = 0.001) between soil depth and 28 functional categories of subsystems level 1. Narrow angles between the vectors of soil depth and a functional category indicate a strong positive relationship, a wide angle, shows a negative relationships, and a 90° angle shows the absence of relationship. Long vectors indicate that the functions are well represented in the plot.

**FIGURE 4:**
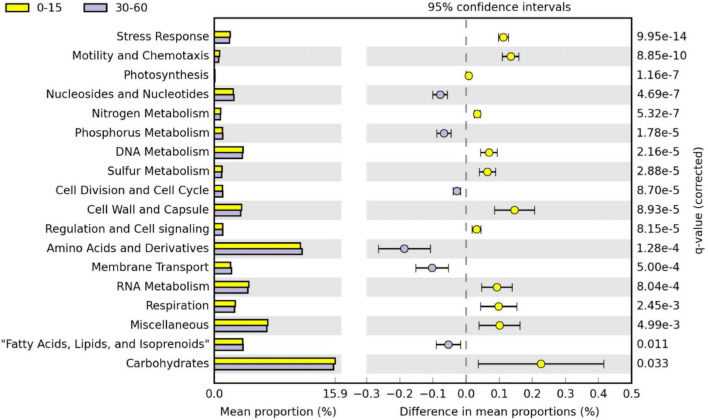
*Post hoc* plot of functional profiles at subsystem level 1 in the 0–15 cm and 30–60 cm soil layers in STAMP. Groups represented in 0–15 cm (yellow) correspond to positive differences between proportions and groups represented in 30–60 cm (purple) correspond to negative differences between proportions. The comparison was accomplished including 15 replicates. Corrected *p*-values were calculated using Benjamini–Hochberg FDR approach (*p* < 0.05). Only significant different functional level1 categories between the 0–15 cm and 30–60 cm soil layers are shown.

Twenty-eight functional categories of subsystem level 1 were detected in all soil layers (Table 1), and 20 of these subsystem level 1 categories were differentially distributed in the soil profile. Genes related to nutrient metabolism [sulfur (S), phosphorus (P), N], DNA and RNA metabolism, “stress response,” “regulation and cell signaling,” “photosynthesis,” “nucleosides and nucleotides,” “motility and chemotaxis,” “cell division and cell cycle,” and “amino acids and derivatives” were strongly stratified in the soil profile (*p* < 0.0001; Table 1). Analysis by RDA indicated that the abundance of soil functional genes was strongly affected by soil depth (*p* = 0.001), and most functional categories were more abundantly distributed at either 0–15-cm or the 30–60-cm depth, with the 15–30-cm soil depth a transition zone (Figure 3). Figure 4, generated by STAMP, shows differences in the functional profiles of subsystem level 1 at the 0–15-cm and 30–60-cm soil depths. More functions were prevalent at 0–15 cm than at 30–60 cm. Only six functions (genes related to nucleosides and nucleotides, P metabolism, cell division and cell cycle, amino acids and derivatives, membrane transport, and fatty acids) were more abundant in the 30–60-cm depth. The other 12 functions with significant differences were more abundant at 0–15-cm depth. Functional genes related to DNA and RNA metabolism were more abundant at 0–15 cm. Similarly, functions related to nutrient metabolism, including N, S, and carbohydrates metabolism, were more abundant in the top depth. Functions related to “stress response,” “photosynthesis,” “N metabolism,” “DNA metabolism,” “S metabolism,” “cell wall and capsule,” “regulation and cell signaling,” “RNA metabolism,” “respiration,” “miscellaneous,” and “carbohydrates” had both higher abundance and higher level of diversity (Shannon *H*′) at 0–15 cm (Figure 4 and Table 2).

**TABLE 2:** Least square means of Shannon index and probability of depth effects on subsystem level 1 functions detected in different soil depths. Values are means (*n* = 15).

Depth	Cell wall and capsule	Functions					
0–15	3.14 a	**Virulence, disease and defense**	**Sulfur metabolism**	**Secondary metabolism**	**RNA metabolism**	**Respiration**	**Regulation and cell signaling**

15–30	3.12 b	2.95 a	2.06 a	2.181 ab	3.03 a	2.73 a	2.42 a
30–60	3.1 b	2.93 b	2.04 b	2.177 a	3.02 b	2.69 b	2.37 b
*p*-Value	**<0.0001**	2.92 c	2.03c	2.154 b	3.02 b	2.66 c	2.36 b
		**<0.0001**	**<0.0001**	**0.031**	**<0.0001**	**<0.0001**	**<0.0001**

	**Protein metabolism**						

0–15	3.99 a	**Photosynthesis**	**Nucleosides and nucleotides**	**Miscellaneous**	**Metabolism of aromatic compounds**	**Membrane transport**	**Dormancy and sporulation**

15–30	3.586 b	0.1 a	2.17	3.61 a	3.044 a	3.175 a	1.17 a
30–60	3.585 b	0.07 b	2.18	3.6 b	3.039 a	3.148 b	1.14 b
p-Value	**<0.0001**	0.06 b	2.17	3.6 b	3.029 b	3.145 b	1.13 b
		**0.001**	0.29	**<0.0001**	**0.003**	**<0.0001**	**0.001**

	**DNA metabolism**						

0–15	2.687 a	**Clustering-based subsystems**	**Amino acids and derivatives**	**Fatty acids, lipids, and isoprenoids**	**Cofactors, vitamins, prosthetic groups, pigments**	**Phages, prophages, transposable elements, plasmids**	**Motility and chemotaxis**

15–30	2.678 b	4.573 a	3.69 a	2.26 a	2.98 a	1.94	1.26
30–60	2.668 c	4.553 b	3.69 a	2.24 b	2.98 ab	1.81	1.26
*p*-Value	**<0.0001**	4.546c	3.68 b	2.23 c	2.97 b	1.82	1.27
		**<0.0001**	**<0.0001**	**<0.0001**	**0.026**	0.08	0.07

	**Cell division and cell cycle**						

0–15	2.081 a	**Iron acquisition and metabolism**	**Stress response**	**Carbohydrates**	**Potassium metabolism**	**Nitrogen metabolism**	**Phosphorus metabolism**

15–30	2.072ab	2.06	2.85 a	3.79 a	0.417 b	1.59 a	1.17 b
30–60	2.071 b	2.05	2.82 b	3.78 b	0.424 b	1.53 b	1.2 a
*p*-Value	**0.014**	2.07	2.8 c	3.77 c	0.504 a	1.49 c	1.2 a

*Different letters within each row and p-values in bold indicate statistically significant differences (ANOVA followed by Tukey HSD; α = 0.05).*

To compare the trend of functions among soil depths, we plotted the relative abundance of the functional profiles in different land use types, as influenced by soil depth. Supplementary Figure 5 shows that functional profiles of “P and K metabolism,” “amino acids and derivatives,” “membrane transport,” “nucleosides and nucleotides,” “cell division and cell cycle,” and “fatty acids, lipids, and isoprenoids” were most important in deeper soil layers in all land use types. However, functional gene abundance often declined with soil depth. These functional genes included nutrient metabolism [N, S, carbohydrates, and zinc (Zn)], “stress response,” “motility and chemotaxis,” “cell wall and capsule,” “DNA metabolism,” “photosynthesis,” “regulation and cell signaling,” and “RNA metabolism” (Supplementary Figure 5). Some functional profiles were not significantly affected by soil depth (e.g., “clustering-based subsystems,” “metabolism of aromatic compounds,” and “protein metabolism”).

### Soil Chemical and Physical Properties and Their Relationship to Community and Functional Profiles

There was a significant effect of depth on bulk density, so abundances of soil nutrients are presented as kg (or mg) ha^–1^ rather than concentration per kg of soil (Table 3 and Supplementary Table 2). There were no significant interactions of depth and land use for any of the soil chemical and physical parameters analyzed. Land use effect was significant for only a few parameters (Supplementary Table 2), with organic C concentrations greater in the pastures than cropland, nitrate concentrations greater in cropland than the pastures, and total N and DTPA-extractable potassium (K) concentrations greater in the seeded pasture compared to cropland.

**TABLE 3:** Soil chemical data by depth. Values are means ± std. err. (*n* = 15).

Property	Soil Depths (cm)			
	0–15	15–30	30–60	*p*-Value
pH (paste)	6.84 ± 0.17*a*	7.14 ± 0.14*b*	7.95 ± 0.06*c*	**<0.0001**
Sand%	40.6 ± 2.47	37.8 ± 2.51	25.7 ± 2.63	0.392
Clay%	26.7 ± 1.22*b*	31.6 ± 1.09*a*	34.3 ± 1.20*a*	**0.002**
Silt%	32.7 ± 1.59	30.6 ± 1.84	30.1 ± 1.93	0.553
Electrical conductivity (dS m^–1^)	0.44 ± 0.06	0.72 ± 0.27	1.67 ± 0.75	0.300
Bulk density (g cm^–1^)	1.16 ± 0.04*b*	1.26 ± 0.0.4*ab*	1.36 ± 0.03*a*	**0.004**
Total carbon (C; Mg ha^–1^)	35.7 ± 2.29*b*	28.2 ± 3.25*c*	86.8 ± 3.81*a*	**<0.0001**
Organic C (Mg ha^–1^)	30.9 ± 2.63*a*	16.5 ± 1.13*b*	26.0 ± 2.71*a*	**<0.0001**
Org C/Total C	0.87 ± 0.04*a*	0.68 ± 0.07*a*	0.37 ± 0.04*b*	**<0.0001**
Total nitrogen (N, Mg ha^–1^)	3.27 ± 0.20*a*	2.14 ± 0.10*b*	3.44 ± 0.23*a*	**<0.0001**
CNratio	0.09 ± 0.01*a*	0.09 ± 0.01*a*	0.04 ± 0.01*b*	**<0.0001**
Nitrate (kg ha^–1^)	3.04 ± 0.45	2.05 ± 0.44	2.77 ± 0.67	0.162
Total phosphorus (P, kg ha^–1^)	911 ± 37.6*b*	911 ± 44.3*b*	2,136 ± 98.4*a*	**<0.0001**
Organic P (kg ha^–1^)	449 ± 30.0	382 ± 55.5	479 ± 48.0	0.179
Olsen P (kg ha^–1^)	9.94.1.69 a	3.40 ± 0.56*b*	8.02 ± 2.75*ab*	**0.006**
Meh P (kg ha^–1^)	38.3 ± 4.3*a*	13.2 ± 2.6*b*	32.5 ± 10.1*ab*	**0.007**
CaCl_2_-P (kg ha^–1^)	5.47 ± 0.34*b*	4.48 ± 0.14*b*	9.86 ± 0.65*a*	**<0.0001**
Meh calcium (Ca, kg ha^–1^)	5,259 ± 1,568*c*	12,569 ± 3,750*b*	42,026 ± 5,139*a*	**<0.0001**
AACa (kg ha^–1^)	3,420 ± 456*c*	5,266 ± 498*b*	13,484 ± 457*a*	**<0.0001**
Meh magnesium (Mg, kg ha^–1^)	901 ± 82.6*c*	1,757 ± 191*b*	6,599 ± 732*a*	**<0.0001**
AAMg (kg ha^–1^)	729 ± 54.3*c*	1,355 ± 136*b*	4,313 ± 452*a*	**<0.0001**
Meh aluminum (Al, kg ha^–1^)	948 ± 115*a*	709 ± 167*a*	102 ± 69*b*	**<0.0001**
Meh iron (Fe, kg ha^–1^)	182 ± 17.3	126 ± 15.9	183 ± 25.4	0.078
DTPA Fe (kg ha^–1^)	75.1 ± 11.7*a*	32.3 ± 4.31*b*	35.5 ± 3.31*b*	**0.003**
Meh manganese (Mn, kg ha^–1^)	147 ± 11.4*a*	92.1 ± 10.0*b*	212 ± 26.7*a*	**0.002**
DTPA Mn (kg ha^–1^)	38.1 ± 4.28*a*	21.5 ± 0.14*b*	7.95 ± 0.06*c*	**0.001**
DTPA potassium (K, kg ha^–1^)	457 ± 37.1*a*	277 ± 40.7*b*	604 ± 79.9*a*	**<0.0001**
DTPA zinc (kg ha^–1^)	4.53 ± 1.59*a*	1.20 ± 0.32*b*	2.40 ± 0.97*ab*	**0.003**
DTPA copper (Cu, kg ha^–1^)	2.29 ± 0.25*b*	2.65 ± 0.17*b*	5.79 ± 0.42*a*	**<0.0001**
CaCl_2_ SO_4_-S (kg ha^–1^)	13.1 ± 2.96	92.8 ± 81.8	781 ± 466	0.089

*Different letters within each row and p-values in bold indicate statistically significant differences (ANOVA followed by Tukey HSD; α = 0.5). AA, ammonium acetate extraction; DTPA, diethylenetriaminepentaacetic acid extraction; Meh, Mehlich extraction. Data were adapted from Cade-Menun et al. (2017).*

There were significant differences among depths for almost every analyzed parameter (Table 3); the exceptions were sand and silt content, electrical conductivity, and nitrate, organic P, Mehlich-extractable iron (Fe), and CaCl_2_-extractable sulfate (SO_4_-S) concentrations. Both bulk density and clay content were greater at 30–60 cm than at 0–15 cm, suggesting reduced porosity but potentially greater moisture retention from clay. Soil pH was also significantly greater at 30–60 cm than 0–15 cm, and was neutral to slightly acidic at the surface (6.84) but alkaline (7.95) at depth. Other parameters that had significantly greater values at 30–60 cm than 0–15 cm were concentrations of total C, total P, CaCl_2_-extractable P, Mehlich calcium (Ca), ammonium acetate (AA)-extractable Ca and magnesium (Mg), and diethylenetriaminepentaacetic acid (DTPA)-extractable copper (Cu). Parameters that were greater at 0–15 cm than 30–60 cm were the ratio of organic C to total C, CN ratio, and concentrations of Mehlich-extractable aluminum (Al), DTPA-Fe, and DTPA-manganese (Mn). Finally, soil parameters with similar values at 0–15 cm and 30–60 cm but values significantly different in the 30–60-cm depth were organic C, total N, Olsen P, Mehlich P, Mehlich Mn, and DTPA-extractable K and zinc (Zn) concentrations. None of the measured soil parameters had significantly greater values at 15–30 cm than 0–15 or 30–60 cm, and this middle depth served as a transition between upper and lower depths for many soil factors. The RDA analysis shows that functional genes for “phages” correlated with organic P concentration (Po; Supplementary Figure 6); functions related to “motility” and “photosynthesis” correlated with organic matter and soil fertility indicators including Fe, Mn, and Zn concentrations; and “iron acquisition” correlated positively with bulk density, and Ca and Mg concentrations, and negatively with Fe concentration.

## Discussion

The taxonomic profiles of the microbial communities and the functional gene profiles on Subsystem Level 1 varied between the 0–15-cm and 30–60-cm soil depths. Our comparisons show that the 15–30-cm depth zone contains overlapping microbial communities and functions and it was considered as an intermediate transition zone between the upper and deeper soil depths. Soil depth influences the structure of the microbial community and the distribution of functional genes. Changes in soil properties and fertility with depth have been linked to shifts in the structure of the soil microbial community in other studies (Davies et al., 2013; Lai et al., 2013; Frank-Fahle et al., 2014; Chen et al., 2015; Wernerehl and Givnish, 2015; Yusuf et al., 2015). Our results show that soil functions at the broad level (subsystem level 1) were stratified in the soil, with most functions concentrated either in the 0–15-cm or 30–60-cm soil depths. These results are particularly important given that (i) ∼50% of root biomass can be below 20 cm (Jackson et al., 1996) and (ii) different crops can take up the same nutrients from different soil depths (Hauggaard-Nielsen et al., 2001). Hence, the distribution of soil microbial communities at different depths could be used to support intercropping. Moreover, understanding the microbial distribution at different soil depths is of relevance for guiding appropriate soil sampling approaches.

According to our metagenomic analysis, the top (0–15 cm) and deeper (30–60 cm) depths are habitats of specific microbial communities carrying distinct functional gene assemblages and functions. Our findings concur with the metagenomics study of Uroz et al. (2013), showing that the level of taxonomic diversity of the soil microbiota seemingly does not decrease with increasing soil depth and is comparable between organic matter-rich (0–10 cm) and mineral (10–20 cm) soil layers. In contrast, other studies report a decline in soil microbial diversity indices in deep soil layers across different land use types and depths (Jumpponen et al., 2010; Will et al., 2010; Upton et al., 2020). Our observations indicate that the structure of the soil’s functional and taxonomic community changes with soil depth. Different patterns of soil microbial taxa and functional gene assemblages exist at different depths in soils in the semiarid region of the North American Great Plains. Compared to the upper- and lower-depth soils, the middle-depth soil had a higher taxonomic diversity compared to the top and deep soil depths.

### Taxonomic Community Structure

The NMDS analysis and the heatmap of phylum/family taxa profiles showed distinct taxonomic community structures at 0–15-cm and 30–60-cm soil depths, while the taxonomic profiles at 15–30 cm shared similarity with profiles of the other depths (Figure 2, Supplementary Figures 2-1,4). The 15–30-cm soil depth of the semiarid prairie ecozone is a taxonomically diverse transition zone. The three soil depths exhibit significant differences in taxonomic abundance at the phylum level, and the Shannon diversity index of the soil microbial community among soil depths supports a comparable diversity in all soil depths, with a slightly higher diversity at the middle soil depth. This result, along with the preferential distribution of phyla at deep or superficial soil depths shows that different microbial taxonomic communities inhabit various soil depths, concomitant with previous reports (Becerra et al., 2014; Jiao et al., 2018). A possible explanation for this change in microbial community structure could be the effect of richness in organic matter (e.g., the ratio of organic C to total C, Table 3) in the top soil depth versus the mineral nature and increased bulk density of the deepest soil depth, resulting in the dominance of the most adapted phyla in each niche. Hence, the middle soil depth is an intermediate milieu where microbial communities from both niches co-exist. This transition zone has the highest diversity index of the three soil layers, reflecting its richness and suitability for various microbial phyla.

Our results indicate that soil depth strongly alters the structure of the microbial community leading to the dominance of different taxa in different soil depths, a conclusion supported by research conducted in various environments (Fierer et al., 2003; Eilers et al., 2012; Feng et al., 2019). In these semiarid prairie soils, we found that the phyla Acidobacteria, Chlorophyta, Bacillariophyta, and Bacteroidetes were concentrated at the top 0–15-cm soil depth while Verrucomicrobia declined below 30 cm (Figure 2). Eilers et al. (2012) have similarly reported that Bacteroidetes declined with soil depth. Further analysis at the family level showed that some families (e.g., Bacillaceae and Listeriaceae) were significantly more abundant at the 0–15-cm depth (Supplementary Figure 4), which is similar to the observation of Davies et al. (2013) of a higher abundance of the family Bacillaceae in a surface silty loam soil in Switzerland. Similarly, Kim et al. (2014) reported large differences in bacterial community structure between the 0–10-cm and the 10–20-cm soil depths in the tundra at Council, Alaska.

### Functions of N Metabolism and Carbohydrates

The prominence of functional genes related to N metabolism and carbohydrates in the surface depth suggests that this important metabolism is more active in the top soil layer than at depth, presumably due to the concentration of organic matter in surface soil by plants (Baisden et al., 2002; Wernerehl and Givnish, 2015; Yusuf et al., 2015), reflected in the higher ratio of organic C to total C in the soils of this study (Table 3). The prominence of N and carbohydrate metabolism genes could reflect differences in the CN ratio with depth (Table 3) but could also be the result of the increased microbial biomass in the surface depth. Phototrophic bacteria are concentrated on the soil surface in response to light (Davies et al., 2013). Studies of native and seeded prairie in other regions have reported that the abundant photosynthetic and N_2_-fixing microphytic crusts covering most open soil surfaces (Knapen et al., 2007) are an important source of available N in the semiarid prairie food chain (Patova et al., 2016). However, these crusts were not specifically observed for the grassland soils of this study (Cade-Menun et al., 2017). Our results are in line with Frank-Fahle et al. (2014) who observed an inverse correlation between N- and C-related functional potential and soil depth. Concomitantly, Wang et al. (2017) found that N-cycling functional genes abundance is significantly inversely proportional to soil depth.

### Functions of P Metabolism, Respiration, and Photosynthesis

In this study, the prominence of many functional genes related to the metabolism of nutrients such as N, S and carbohydrates declined with increasing soil depth, but genes related to P metabolism showed an inverse trend. The functional profile of P metabolism in the three land use types was significantly proportional to soil depth. Few studies have documented the relationship between soil P and depth (Van Der Wal et al., 2007; Eriksson et al., 2016; Cade-Menun et al., 2017). In the soils of this study, total P was significantly greater at the 30–60-cm depth than the other depths, but soil test P concentrations (Olsen P, Mehlich P) were significantly greater at the soil surface and there was no significant difference in organic P concentrations with depth (Table 3). Greater soil test P concentrations indicate that soil P was more labile at the soil surface than deeper in the soil profile where P would be more tightly bound to Ca and Mg at higher pH, which may explain the increase in genes related to P metabolism in the lower depths. The correlation of organic P with functional genes for “phages” in the RDA analysis (Supplementary Figure 6) probably points toward the acquisition of P cycling genes through phage-mediated horizontal gene transfer.

The abundance of functional genes related to respiration decreased with increasing soil depth. Other studies indicated that soil respiration was mainly influenced by soil temperature (Yu et al., 2011), organic matter and water (Lai et al., 2013), and selected plant species (Dias et al., 2010). Moreover, soil respiration is a functional pathway of the C cycle (Dias et al., 2010; Buck and St Clair, 2012). Considering the conditions of the soil environment in our study, decreasing respiration with soil depth is most likely due to decreasing soil organic matter concentrations with soil depth, as concluded by previous studies (Fang and Moncrieff, 2005; Wang et al., 2019). This is reflected in the decreasing ratio of organic C to total C, and increasing clay content and bulk density in these prairie soils (Table 3). The soil functional gene profile related to photosynthesis strongly declined with soil depth, an understandable trend since light can only reach a very thin depth in soil and only affects the phototrophic communities in this thin top soil layer (Jeffery et al., 2009; Davies et al., 2013). Functions related to “motility” and “photosynthesis” were also correlated with organic matter and soil fertility indicators including Fe, Mn, and Zn concentrations. Furthermore, the functional gene profiles related to photosynthesis decreased more sharply in cropland compared to the two prairie land types (Supplementary Figure 5), a noteworthy observation given the importance of phototrophic communities in pesticide degradation on the soil surface (Davies et al., 2013).

## Conclusion

The application of metagenomic analysis gave new insights into soil microbial community structure. The results of this study show, for the first time, distinctive soil microbial communities and functional structures at different soil depths in a semiarid prairie agroecosystem. Phyla Bacteroidetes, Chlorophyta, Bacillariophyta, and Acidobacteria were more abundant at the soil surface, while the abundance of phyla Ascomycota, Dictyoglomi, Aquificae, and Planctomycetes increased at 30–60-cm soil depth. Functional genes related to N and carbohydrates metabolisms were prominent in the top soil layer, while P metabolism genes were prominent in deeper soil layers, and were related to soil chemical properties. We conclude that there are specific patterns of microbial communities at different soil depths with the middle soil depth representing a diverse, intermediate phase of co-existence among various taxonomic phyla.

## Data Availability Statement

HiSeq data are available through MG-RAST at the following MG RAST id : 4569676.3, 4571346.3, 4569677.3, 4569678.3, 4569575.3, 4569576.3, 4569681.3, 4569682.3, 4569683.3, 4569688.3, 4569689.3, 4569690.3, 4569692.3, 4569693.3, 4569694.3, 4569578.3, 4571354.3, 4569698.3, 4569703.3, 4569704.3, 4569705.3, 4569579.3, 4571360.3, 4569580.3, 4568990.3, 4569041.3, 4569067.3, 4569044.3, 4569583.3, 4572094.3, 4572095.3, 4569045.3, 4569046.3, 4572096.3, 4569588.3, 4572097.3, 4572100.3, 4571355.3, 4569049.3, 4569593.3, 4569594.3, 4572671.3, 4569050.3, 4569051.3, and 4569052.3.

## Author Contributions

ZR and MD: data analyses and manuscript writing. CH, LB, BC-M, MS-A, and MH: conceptualization, experimental design, supervision of field experiments, and manuscript editing. YT: bioinformatics analyses. CH, MS-A, and MH: concepts, design, supervision, and contribution to the manuscript writing. All authors contributed to the article and approved the submitted version.

## Conflict of Interest

MD was employed by Research and Development of Enterra Corporation. The remaining authors declare that the research was conducted in the absence of any commercial or financial relationships that could be construed as a potential conflict of interest.

## Publisher’s Note

All claims expressed in this article are solely those of the authors and do not necessarily represent those of their affiliated organizations, or those of the publisher, the editors and the reviewers. Any product that may be evaluated in this article, or claim that may be made by its manufacturer, is not guaranteed or endorsed by the publisher.

## Footnotes

1
http://metagenomics.anl.gov/

2
http://rdp.cme.msu.edu/index.jsp
