# Supplementary material for: Soil Depth Significantly Shifted Microbial Community Structures and Functions in a Semiarid Prairie Agroecosystem
Source: Front Microbiol. 2022 Jun 8;13:815890. doi: 10.3389/fmicb.2022.815890 (PMC9213743; doi:10.3389/fmicb.2022.815890)
Supplement: Supplementary file 1 [file Data_Sheet_1.docx]

**Supplementary Material**

Soil Depth Significantly Shifted Microbial Community Structures and Functions in a Semiarid Prairie Agroecosystem

Zineb Rchiad, Mulan Dai, Chantal Hamel, Luke D. Bainard, Barbara J. Cade-Menun, Yves Terrat, Marc St-Arnaud and Mohamed Hijri

Number of pages: 11

Number of Tables: 2

Number of Figures:6

**Sample Collection and Analysis**

The samples in this study were collected as part of a larger land use study; see Cade-Menun et al. (2017) for a detailed description. Sample collection is described in the main text of this manuscript, with cores sectioned into six depths (0-7.5, 7.5-15, 15-30, 30-60, 60-90, and 90-120 cm). Soil samples were transported on ice to the laboratory, and stored at 4°C overnight. Samples for metagenomic analysis were removed, another subsample was used to determine bulk density adjusted for stoniness and the remainder was air-dried, ground and sieved to < 2 mm, and pooled to produce one sample for each depth per land use at each location.

Particle size analysis to determine soil texture was done with the hydrometer method (Gee and Bauder 1986). Soil pH and electrical conductivity (EC) were measured in water saturation paste (Hendershot et al. 2008) and paste extracts (Miller and Curtin 2008). Soils were analyzed for total carbon (C), total nitrogen (N_t_), and organic C (after acidification with HCl; C_Org_) by dry combustion (Vario Micro Cube, Elementar), and for total phosphorus (P) by digestion (Parkinson and Allen 1975; O’Halloran and Cade-Menun 2008) followed by colorimetric analysis (Murphy and Riley 1962). Total organic P was determined by the ignition method followed by colorimetric analysis (Saunders and Williams 1955; O’Halloran and Cade-Menun 2008). Mehlich-extractable nutrients were determined by shaking 2.5 g of soil with 25 ml of Mehlich-3 solution (pH 2.3) for 5 min (Mehlich 1984) and the concentrations of P, aluminum (Al), iron (Fe), calcium (Ca), magnesium (Mg) and manganese (Mn) were determined with inductively coupled plasma optical emission spectroscopy (ICP-OES Thermo Scientific ICAP 6300 Duo). Sodium bicarbonate extraction, followed by colorimetric analysis using a Technicon Autoanalyzer, was used to determine nitrate (NO_3_), potassium (K) and Olsen P (Gentry and Willis 1988; Hamm et al. 1970). The concentrations of Fe, Mn, copper (Cu) and zinc (Zn) were determined by ICP-OAS in DTPA (diethylenetriaminepentaacetic acid) extracts (Lindsay and Norvell 1978). Exchangeable Ca and Mg were extracted in ammonium acetate (AA) and measured by ICP-OAS (Hendershot et al. 2008). Dilute salt-extractable P (CaCl_2_-P) and sulfate (SO_4_-S) were determined in 0.01 M CaCl_2_ extracts followed by colorimetric analysis (Hamm et al. 1973; Self-Davis et al. 2009).

The results for all land use types and all depths are presented in Cade-Menun et al. (2017). To match the samples and depths for the metagenomic data in the current study, the data for the 0-7.5- and 7.5-15-cm depths were averaged together, the 60-90- and 90-120 cm depths were excluded, and data from only three of the four sampled land use types were used (native prairie, tame (seeded) prairie and cropland). The normality of data was tested by fitting to a normal distribution and assessing the goodness of fit with a Shapiro-Wilks w test. If needed, data were transformed prior to statistical analysis by log (n +0.5), but means and standard errors in Table 3 and Supplementary Table S1 were calculated from untransformed data. Statistical analyses were performed with the statistical package JMP (version 4.04, SAS Institute, Inc.), with significance set at *α =* 0.05. The Tukey’s Highest Significant Difference (HSD) test was used to compare means.Means among land use types were compared with two-factor ANOVA using land use, depth and the land use x depth interaction. The interaction was not significant; data means by depth with all land use types together are shown in Table 3 in the main paper (n=15), while those by land use with all depths together are shown in Supplemental Fig. S1.

**Sequencing data**

HiSeq data are available through MG-RAST at the following MG RAST id : 4569676.3, 4571346.3, 4569677.3, 4569678.3, 4569575.3, 4569576.3, 4569681.3,  4569682.3, 4569683.3, 4569688.3, 4569689.3, 4569690.3, 4569692.3, 4569693.3, 4569694.3, 4569578.3, 4571354.3, 4569698.3, 4569703.3, 4569704.3, 4569705.3, 4569579.3, 4571360.3, 4569580.3, 4568990.3, 4569041.3, 4569067.3, 4569044.3, 4569583.3,  4572094.3, 4572095.3, 4569045.3, 4569046.3, 4572096.3, 4569588.3, 4572097.3, 4572100.3, 4571355.3, 4569049.3, 4569593.3, 4569594.3, 4572671.3, 4569050.3, 4569051.3, 4569052.3.

**References**

Cade-Menun, B.J., Bainard, L.D., LaForge, K., Schellenberg, M., Houston, B., Hamel, C. 2017. Long-term agricultural land use affects chemical and physical properties of soils from Southwest Saskatchewan. Canadian Journal of Soil Science 97, 650-666.

Gee, G.W., Bauder, J.W. Particle size analysis. 1986. Pages 383-411 *in*Klute, A., ed. Methods of Soil Analysis Part 1, 2^nd^ Edition. American Society of Agronomy - Soil Science Society of America.

Gentry, C. E., Willis, R. B. 1988. Improved method for automated determination of ammonium in soil extracts. Communications in Soil Science and Plant Analysis 19, 721-737.

Hamm, J.W., Radford, F.G., Halstead, E.H. 1970. The simultaneous determination of nitrogen, phosphorus and potassium in sodium bicarbonate extracts of soils. In: Technicon International Congress, Advances in Automatic Analysis. Industrial Analysis, Vol. II: 65-69.

Hamm, J.W., Bettany, J.R., Halstead, E.H. 1973. A soil test for sulphur and interpretive criteria for Saskatchewan. Communications in Soil Science and Plant Analysis 4, 219-231.

Hendershot, W.H., Lalande, H., Duquette, M. 2008. Soil reaction and exchangeable acidity. Pages 173–178*in* Carter, M.R., Gregorich, E.G., eds. Soil Sampling and Methods of Analysis, 2^nd^ Edition. Canadian Society of Soil Science and CRC Press.

Lindsay, W.L., Norvell, W.A. 1978. Development of a DTPA soil test for zinc, iron, manganese and copper. Soil Science Society of America Journal 42, 421-428.

Mehlich, A. 1984. Mehlich 3 soil test extractant: A modification of Mehlich 2 extractant. Communications in Soil Science and Plant Analysis 15, 1409-1416.

Miller, J.J., Curtin, D. 2008. Electrical conductivity and soluble ions. Pages 161-171*in* Carter, M.R., Gregorich,E.G., eds. Soil Sampling and Methods of Analysis, 2^nd^ Edition. Canadian Society of Soil Science and CRC Press.

Murphy, J., Riley, J.P. 1962. A modified single solution method for determination of phosphate in natural waters. Analytica ChimicaActa 27, 31-36.

O’Halloran, I.P., Cade-Menun, B.J. 2008. Total and organic phosphorus. Pages 265-291 i*n* Carter, M.R., Gregorich,E.G., eds. Soil Sampling and Methods of Analysis, 2^nd^ Edition. Canadian Society of Soil Science and CRC Press.

Parkinson, J.A., Allen S.E. 1975. A wet oxidation procedure suitable for the determination of nitrogen and mineral nutrients in biological material. Communications in Soil Science and Plant Analysis 6, 1-11.

Saunders, W., Williams, E. 1955. Observations on the determination of total organic phosphorus in soils. Journal of Soil Science 6, 254-267.

Self-Davis, M.C., Moore Jr., P. A., and Joern, B.C. 2009. Water-or dilute salt-extractable phosphorus in soil. Pages 22-24 *in* Kovar, J.L. Pierzynski,G.M., eds. Methods of Phosphorus Analysis, 2^nd^ Edition. Southern Cooperative Series Bulletin

**Supplementary Table S1.** Relative abundance of all detected phyla in three different soil depths.

| Domain | Phylum | Soil Depths (cm) | | | p-values (corrected) |
| --- | --- | --- | --- | --- | --- |
|  |  | 0-15 | 15-30 | 30-60 |  |
| Bacteria | Actinobacteria | 28.943 | 28.983 | 31.702 | 0.192 |
| Bacteria | unclassified (derived from Bacteria) | 26.896 | 26.650 | 26.585 | 0.964 |
| Bacteria | Proteobacteria | 12.253 | 12.775 | 12.363 | 0.356 |
| Bacteria | Firmicutes | 7.273 | 7.749 | 8.030 | 0.447 |
| Bacteria | Verrucomicrobia | 5.740 | 5.272 | 2.685 | 0.004 |
| unassigned | unassigned | 4.341 | 4.228 | 4.396 | 0.598 |
| Bacteria | Bacteroidetes | 3.663 | 2.171 | 2.003 | 0.000 |
| Bacteria | Chloroflexi | 1.400 | 1.495 | 1.530 | 0.451 |
| Bacteria | Gemmatimonadetes | 1.380 | 1.434 | 1.573 | 0.695 |
| Bacteria | Planctomycetes | 1.355 | 1.517 | 1.684 | 0.030 |
| Eukaryota | Streptophyta | 1.226 | 1.360 | 1.281 | 0.433 |
| Bacteria | Acidobacteria | 1.115 | 0.760 | 0.426 | 0.012 |
| Archaea | Thaumarchaeota | 0.805 | 0.927 | 0.741 | 0.396 |
| Bacteria | Cyanobacteria | 0.640 | 0.877 | 0.854 | 0.034 |
| unclassified seq. | unclassified (derived from unclassified sequences) | 0.616 | 0.544 | 0.611 | 0.441 |
| Eukaryota | Ascomycota | 0.562 | 0.901 | 1.182 | 0.000 |
| Bacteria | Nitrospirae | 0.248 | 0.555 | 0.623 | 0.000 |
| Bacteria | Thermotogae | 0.224 | 0.263 | 0.264 | 0.386 |
| Bacteria | Dictyoglomi | 0.181 | 0.232 | 0.316 | 0.008 |
| Bacteria | Spirochaetes | 0.172 | 0.178 | 0.132 | 0.228 |
| Bacteria | Chlamydiae | 0.142 | 0.130 | 0.075 | 0.097 |
| Archaea | Crenarchaeota | 0.137 | 0.171 | 0.085 | 0.004 |
| Bacteria | Tenericutes | 0.126 | 0.166 | 0.221 | 0.053 |
| Eukaryota | unclassified (derived from Eukaryota) | 0.089 | 0.110 | 0.078 | 0.192 |
| Eukaryota | Basidiomycota | 0.080 | 0.071 | 0.075 | 0.971 |
| Bacteria | Chlorobi | 0.069 | 0.102 | 0.118 | 0.081 |
| Eukaryota | Chlorophyta | 0.057 | 0.037 | 0.018 | 0.004 |
| Bacteria | Deinococcus-Thermus | 0.052 | 0.077 | 0.105 | 0.054 |
| Eukaryota | Bacillariophyta | 0.035 | 0.018 | 0.005 | 0.005 |
| Bacteria | Aquificae | 0.033 | 0.063 | 0.082 | 0.013 |
| Archaea | Euryarchaeota | 0.031 | 0.031 | 0.029 | 0.992 |
| Bacteria | Synergistetes | 0.027 | 0.021 | 0.003 | 0.112 |
| Bacteria | Thermodesulfobacteria | 0.021 | 0.031 | 0.028 | 0.466 |
| Bacteria | Fusobacteria | 0.016 | 0.018 | 0.035 | 0.188 |
| Bacteria | Fibrobacteres | 0.014 | 0.021 | 0.013 | 0.421 |
| Bacteria | Deferribacteres | 0.013 | 0.027 | 0.019 | 0.355 |
| Archaea | unclassified (derived from Archaea) | 0.011 | 0.016 | 0.013 | 0.824 |
| Archaea | Korarchaeota | 0.007 | 0.009 | 0.006 | 0.810 |
| Eukaryota | Eustigmatophyceae | 0.001 | 0.002 | 0.001 | 0.963 |
| Eukaryota | Phaeophyceae | 0.001 | 0.002 | 0.000 | 0.442 |
| Bacteria | Elusimicrobia | 0.001 | 0.003 | 0.010 | 0.061 |
| Eukaryota | Euglenida | 0.001 | 0.004 | 0.002 | 0.435 |
| Eukaryota | Pinguiophyceae | 0.000 | 0.000 | 0.000 | 1.000 |

**Supplementary Table S2**. Soil chemical data by land use.Values are means ± std, err. (n=15). Different letters within each row indicate statistically significant differences (ANOVA followed by Tukey HSD; α=0.5). AA, ammonium acetate extraction; DTPA, diethylenetriaminepentaacetic acid extraction;Meh, Mehlich extraction.

| **Property** | **Land Use** | | | |
| --- | --- | --- | --- | --- |
|  | **Cropland** | **Native Pasture** | **Seeded Pasture** | **P > F** |
| **pH (paste)** | 7.49±0.21 | 7.01±0.20 | 7.08±0.20 | 0.201 |
| **Sand %** | 35.4±3.09 | 40.5±2.01 | 38.2±2.39 | 0.369 |
| **Clay** % | 31.8±1.41 | 30.7±1.34 | 30.0±1.52 | 0.660 |
| **Silt** % | 32.8±1.95 | 28.8±1.46 | 31.8±1.83 | 0.255 |
| **Electrical conductivity**(dS m^-1^) | 0.40±0.03 | 0.89±0.45 | 1.54±0.67 | 0.194 |
| **Bulk density**(g cm^-1^) | 1.36±0.04 | 1.18±0.05 | 1.24±0.02 | 0.068 |
| **Total carbon**(C;Mg ha^-1^) | 51.5±7.97 | 48.3±36.85 | 50.9±8.04 | 0.982 |
| **Organic C (**Mg ha^-1^) | 18.4±1.82b | 27.2±2.62 a | 27.7±3.02 a | **0.009** |
| **Org C/Total C** | 0.49±0.08 | 0.67±0.07 | 0.70±0.08 | 0.129 |
| **Total nitrogen**(N,Mg ha^-1^) | 2.50±0.19b | 3.11±0.22ab | 3.25±0.26 a | **0.009** |
| **CN ratio** | 0.07±0.01 | 0.08±0.01 | 0.08±0.01 | 0.303 |
| **Nitrate (**kg ha^-1^) | 4.39±0.65 a | 1.48±0.17 b | 1.98±0.29 b | **<0.001** |
| **Total phosphorus**(P,kg ha^-1^) | 1322±145 | 1266±176 | 1370±179 | 0.817 |
| **Organic P (**kg ha^-1^) | 352±25.1 b | 412±37.2 ab | 545±56.8 a | **0.006** |
| **Olsen P (**kg ha^-1^) | 6.34±1.96 | 5.97±1.98 | 9.04±2.04 | 0.196 |
| **Meh P (**kg ha^-1^) | 22.0±6.18 | 27.9±6.98 | 34.1±7.82 | 0.353 |
| **CaCl_2_-P (**kg ha^-1^) | 6.27±0.74 | 6.66±0.81 | 6.87±0.81 | 0.855 |
| **Meh calcium**(Ca,kg ha^-1^) | 19193±3734 | 19856±6981 | 20804±5855 | 0.619 |
| **AA Ca (**kg ha^-1^) | 8389±1130 | 7084±1357 | 6697±1239 | 0.358 |
| **Meh magnesium**(Mg,kg ha^-1^) | 3022±753 | 3050±685 | 3184±943 | 0.960 |
| **AA Mg (**kg ha^-1^) | 2137±467 | 2179±465 | 2082±561 | 0.943 |
| **Meh aluminum**(Al,kg ha^-1^) | 429±146 | 622±160 | 708±153 | 0.555 |
| **Meh iron (**Fe, kg ha^-1^) | 167±20.3 | 158±16.2 | 166±26.0 | 0.952 |
| **DTPA Fe (**kg ha^-1^) | 40.9±8.84 | 49.7±8.16 | 82.4±9.92 | 0.380 |
| **Meh manganese (**Mn, kg ha^-1^) | 178±26.4 | 138±20.2 | 134±16.4 | 0.435 |
| **DTPA Mn (**kg ha^-1^) | 23.2±4.88 | 24.5±2.40 | 29.8±4.16 | 0.384 |
| **DTPA potassium (**K, kg ha^-1^) | 336±37.9 b | 442±45.7 ab | 560±89.0 a | **0.048** |
| **DTPA zinc (**kg ha^-1^) | 3.89±1.88 | 2.48±0.39 | 1.77±0.37 | 0.581 |
| **DTPA copper**(Cu,kg ha^-1^) | 3.45±0.41 | 3.34±0.53 | 3.95±0.57 | 0.631 |
| **CaCl_2_SO_4_-S (**kg ha^-1^) | 13.2±1.96 | 339±327 | 535±364 | 0.287 |

**Supplementary Figure S1**


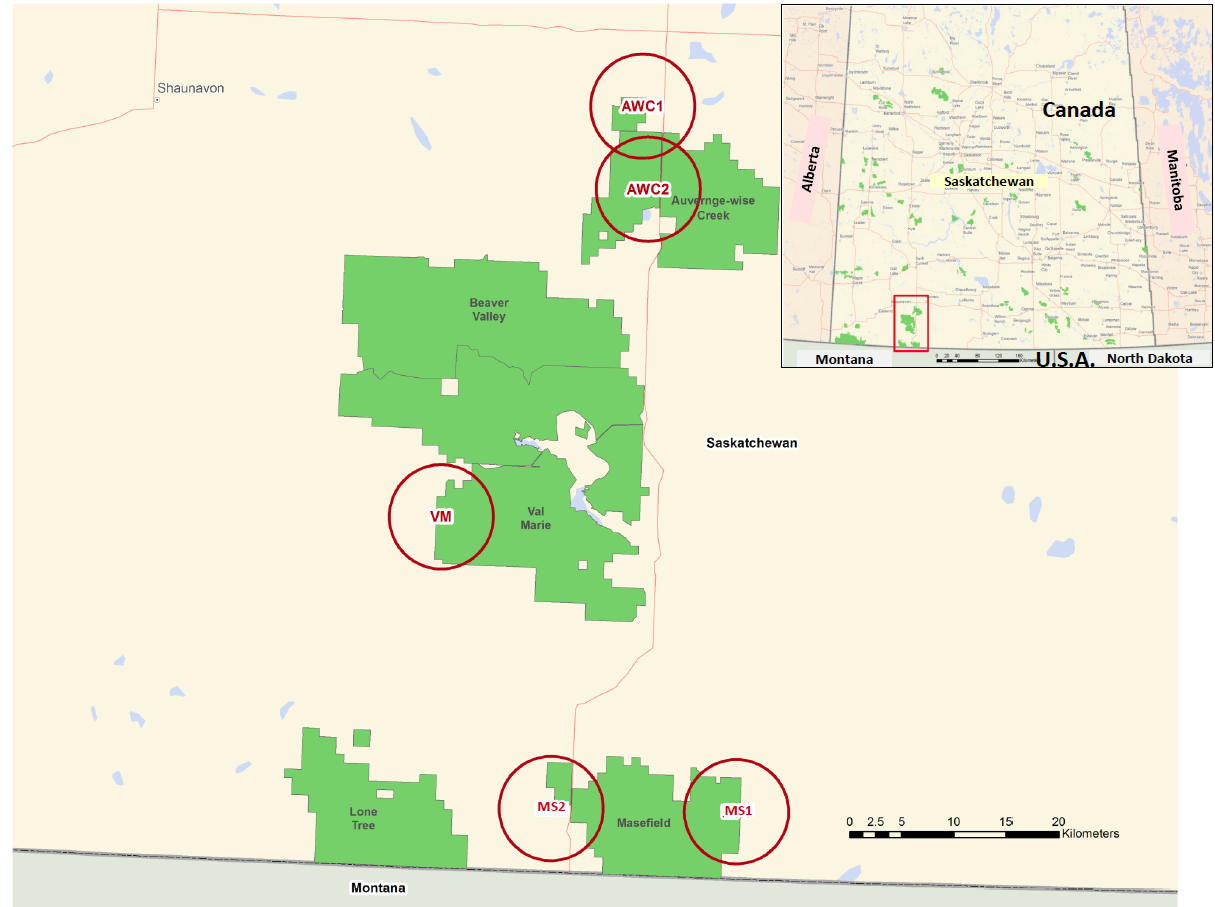


Supplementary Figure S1. Locations of the five sets of sampling sites in southwestern Saskatchewan, in the North American Great Plains. Three community pastures, Auvergne–Wise Creek, Val Marie, and Masefield, were used is this study. Figure adapted from Cade-Menun et al. (2017).

**Supplementary Figure S2**


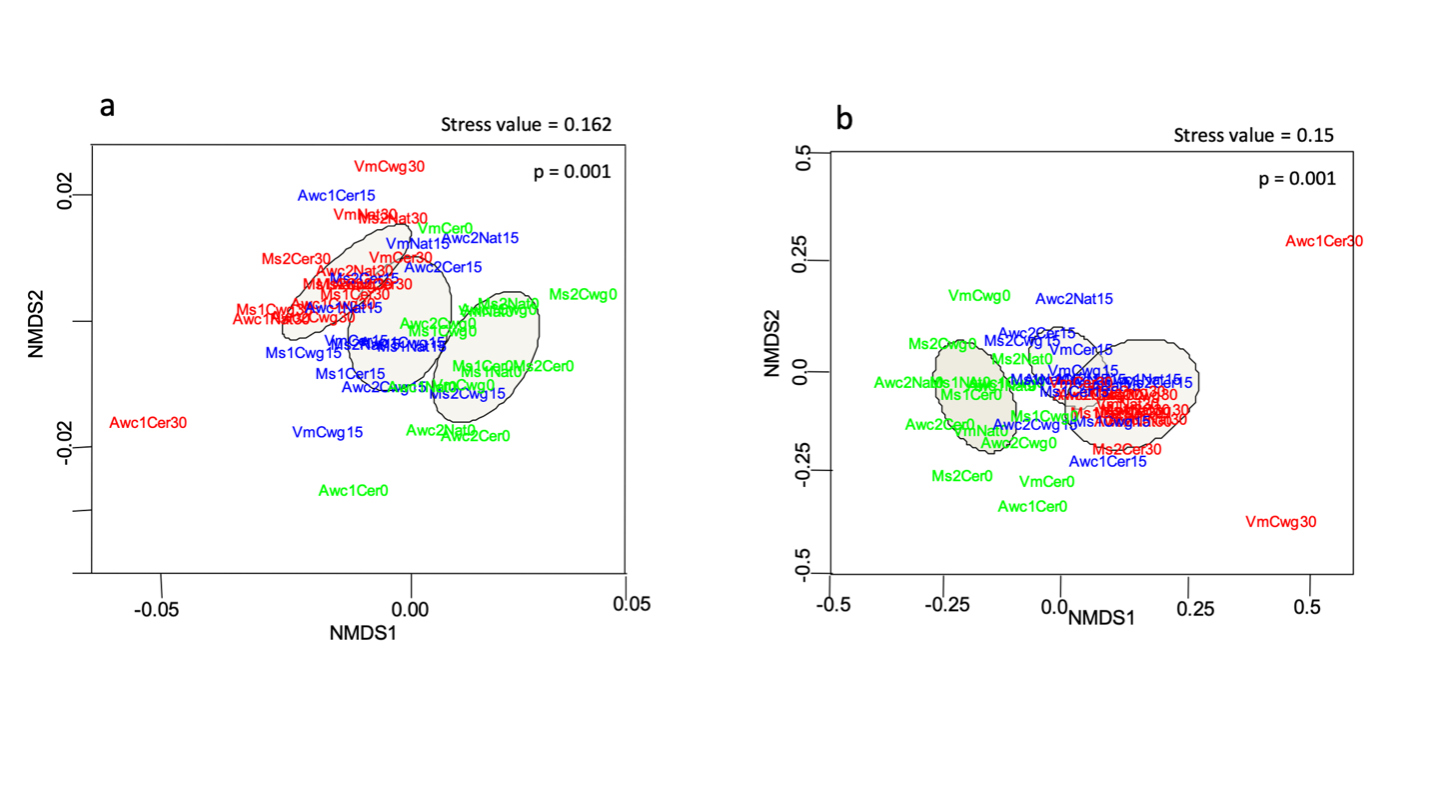


Supplementary Figure S2. NMDS plot of Bray-Curtis distances in the soil microbial community-by-site matrix related to 45 samples located in southwest Saskatchewan. Functional (a) and taxonomic (b) analyses using relative abundance of functional and taxonomic units based on SEED at subsystems level1 and RDP database matches at the family level. The 45 data labels indicate location, land use type, and soil depth; soil depth is color coded. The data cluster by soil depth based on similarity index, as highlighted by ellipses. Stress values are shown in the upper right of the graphs. Stress > 0.05 provides an excellent representation of the data in reduced dimensions, > 0.1 is great, > 0.2 is good, and stress > 0.3 provides a poor representation of the data.

**Supplementary Figure S3**

Supplementary Figure S3. Principle component analysis (PCA) of functional profiles in three soil depths. Depicted are the first two components (PC1 and PC2) of the principle component analysis generated by STAMP, which is based on the relative abundance of the functional sequences on subsystems level 1, processed by MG-RAST.

**Supplementary Figure S4**

Supplementary Figure S4. Heatmap comparing the taxonomic profiles (on family level) in three soil layers, which shows relative abundance of families averaged by each soil depth (*n* = 15), the deeper blue cell, the higher relative abundance of family. Differences in functions among soil depths are indicated by *P* values < 0.05.

**Supplementary Figure S5**

Supplementary Figure S5. Relative abundance of functional genes in different soil layers. The abundance of each functional gene is the sum of the functional profiles on subsystems level3 that belongs to the level1 category in each soil depth of each land use types (*n* = 5).

**Supplementary Figure S6**


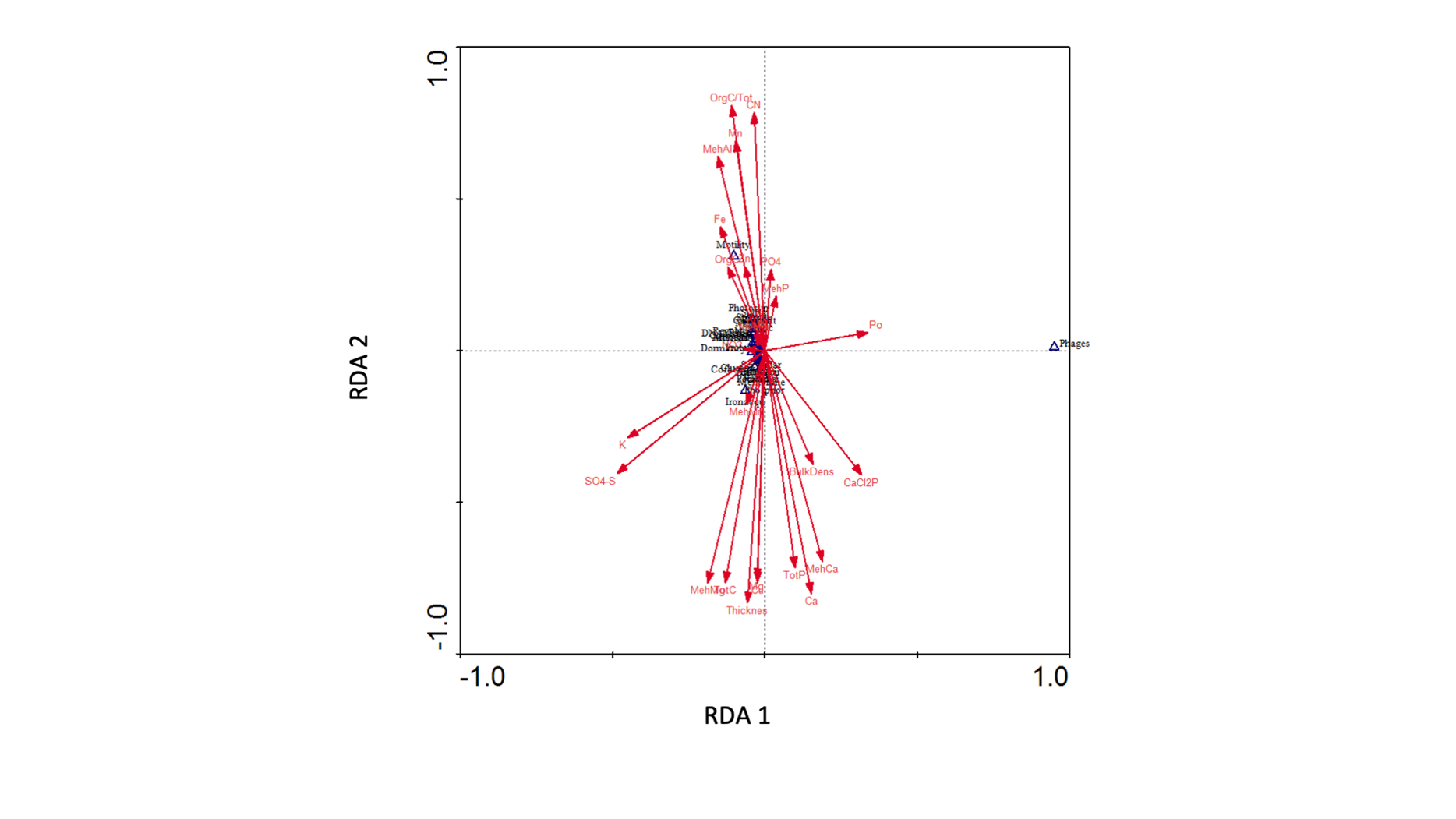


Supplementary Figure S6. RDA showing the relationship between soil properties and 28 functional categories of subsystems level 1. Narrow angles between the vectors of soil depth and a functional category indicate a strong positive relationship, a wide angle, shows a negative relationships, and a 90° angle shows the absence of relationship. Long vectors indicate that the functions are well represented in the plot.
